# Cocaine

**Published:** 1885-10

**Authors:** Walter W. Allport

**Affiliations:** Chicago, Ill.


ARTICLE VIII.

### COCAINE.

WALTER W. ALLPORT, M. D., D. D. S., OF CHICAGO, ILL.

The introduction of cocaine as a local anæsthetic, and the more general use of peroxide of hydrogen ($H_2O_2$) in the treatment of dental and oral diseases, are the principal advance made in the medical department of this practice during the year for which this report is made.

The two forms of cocaine which have been most generally used in surgery are the hydrochlorate and the oleate.

In operations in the mouth, involving the mucous membranes, together with the immediately subjacent tissues, these preparations have proven so efficient there is little question of its value as a local anæsthetic in such cases. But its action on deeper structures, such as involve the roots of teeth, is so uncertain as to render its practicable benefits questionable in the operation of extraction. In the surgical treatment of pockets caused by pyorrhea alveolaris, the anæsthetic effect of this agent is often so great as to render this sometimes very painful operation comparatively painless, and its employment in such cases should rarely be dispensed with. In the treatment of hypersensitive dentine, as well as in the removal of tooth-pulps, its action as an anæsthetic has, under some circumstances, seemed to be all that could be desired. But in far the greater number of cases it has proved of little practical value. More recently, however, a new form of cocaine, known as the citrate, has been introduced in Germany by

Merck, and is now being manufactured by McKesson & Robbins, of New York. In a series of experiments, conducted by Dr. John S. Marshall, of Chicago, it has been shown that for operations on sub-mucous tissues, or in the extraction of teeth, it seems to possess no special advantages over the preparations previously named. But when applied to dentine or the pulp, its action—though not always positive—seems to be more reliable, especially on the dentine, and gives promise of better results. Under favorable conditions it produces anæsthesia of the parts in from five to ten minutes, and the duration of the effect is of sufficient length to afford time for the preparation of the cavity. This effect has, in some cases been prolonged for more than an hour. The pulp has been extirpated without pain after the drug has been applied in from three to twelve minutes.

If the citrate of cocaine be kept in solution for more than three or four days it decomposes and loses its active properties. As introduced by Mr. Merck for dental purposes, it is made into pills by incorporating it with gum tragacanth dissolved in glycerine, each pill containing ⅛ grain of the citrate. In this form it keeps well. A pill is applied to the sensitive cavity and covered with a cotton pledget, moistened in tepid water. It should be allowed to remain from five to twelve minutes, when—if at all—the desired result is produced. In twenty per cent. of the cases where this remedy has been employed it has proven unsuccessful, but it is hoped that this percentage will be reduced by a better knowledge of the drug and the improved methods of its preparation and use.

With this in view, and at the suggestion of Dr. Marshall, McKesson & Robbins are now manufacturing granules containing one-sixteenth of a grain of the citrate of cocaine, without glycerine or any other saccharine excipient, so that the obtundent may act more promptly than it can in the presence of sugar.—*Address at American Medical Association.*

